# Sumatriptan Induced Brugada Syndrome, a Rare Presentation of a Commonly Used Drug

**DOI:** 10.1002/ccr3.70417

**Published:** 2025-06-03

**Authors:** Phool Iqbal, Saba Nabavi Monfared, Daniel Hernan Sacoto, Mustafa Bilal Ozbay, Wael Abdelmottaleb, Valentina Turbay Caballero, Muhammad Khalid Tahir, Savi Mushiyev

**Affiliations:** ^1^ Department of Internal Medicine New York Medical College/Metropolitan Hospital Center New York New York USA; ^2^ Hamad Medical Corporation Emergency and Trauma Clinical Pharmacy Department Doha Qatar; ^3^ Department of Internal Medicine Advocate Christ Medical Center Chicago Illinois USA; ^4^ Department of Cardiology New York Medical College/Metropolitan Hospital Center New York New York USA

**Keywords:** arrhythmia, Brugada syndrome, migraine headaches, sumatriptan

## Abstract

We aim to highlight an important and rare association of Brugada Syndrome with a commonly used medication, Sumatriptan, which is used as an aborting Migraine headaches. This life‐threatening condition needs early diagnosis and prompt management with vigilant follow‐up for better patient outcomes.

## 1 Introduction

Brugada Syndrome (BrS) is a rare inherited autosomal dominant arrhythmogenic channelopathy [1]. It is related to genetics, electrolyte imbalances, drugs, fever, medications, and other components yet to be discovered [1]. It carries an increased risk of progression to ventricular fibrillation (VF) and sudden cardiac death (SCD) [1, 2, 3]. We present a rare case of a symptomatic patient with Brugada type 1 pattern triggered after taking sumatriptan.

## 2 Case History/Examination

A 28‐year‐old male with a past medical history of chronic migraines and anxiety disorder presented with episodic palpitations for the last 2 years. The palpitations were sporadic in nature, self‐resolving, nonexertional, mostly at rest, with an average duration of approximately 3 min. It was associated with a nonradiating left‐sided chest pain of mild to moderate intensity, mild shortness of breath, and dizziness without any syncopal episodes. He had no relevant family history of SCD or cardiac arrhythmia, illicit drug abuse, cigarette smoking, or excessive alcohol consumption. He was taking sumatriptan 50 mg 2–3 times per week intermittently for migraine headaches or sometimes more depending on the migraine attacks.

**FIGURE 1:**
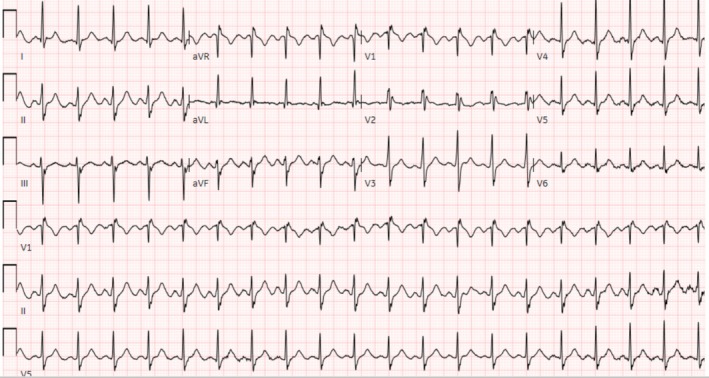
Incomplete right bundle branch pattern.

**FIGURE 2:**
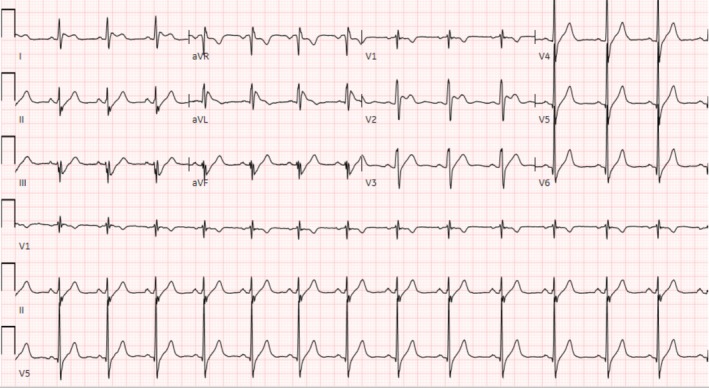
Brugada pattern type II.

Retrospective review of the medical records showed an emergency department visit for migraine exacerbation where EKG showed an incomplete right bundle branch block (RSR′ pattern in V1‐3 QRS complex with 103 s duration) (Figure 1). After providing acute symptomatic relief, the patient was discharged with the continuation of previous migraine medications with outpatient follow up with the neurology and primary care physician. Since the patient did not report any cardiac symptoms therefore no further work up was done for the EKG findings. Three months later, he presented to the ED with palpitations and initial EKG showed a coved and saddleback pattern on V1 and V2, respectively, consistent with Brugada pattern type II (Figure 2). The patient was discharged with a 14‐days Holter monitoring, which did not show arrhythmogenic pattern. During this time period he was seen by the neurologist and was started on amitriptyline 25 mg at night while sumatriptan was discontinued due to dizziness and palpitations. Seven months later, he presented again, complaining of palpitation, this time with more frequent episodes.

## 3 Methods (Differential Diagnosis, Investigations and Treatment)

**FIGURE 3:**
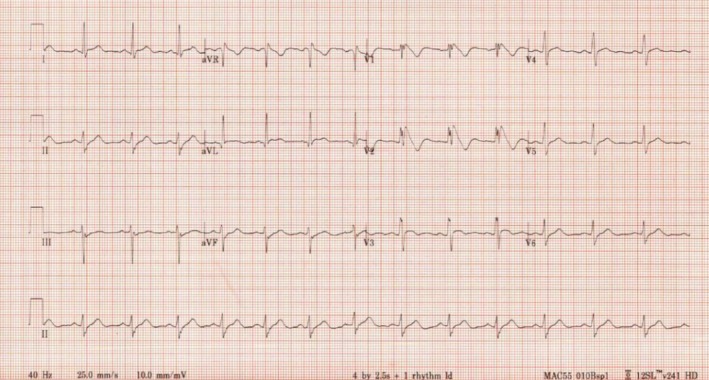
Brugada pattern type I.

Based on the patient's history and further clinical assessment and accordingly laboratory and imaging studies ruled out differential diagnosis like illicit substance use like cocaine etc., alcohol use disorder associated cardiomyopathy, acute coronary syndrome and congenital heart defect, acute coronary syndrome was ruled out since his age, typical chest pain, He has been hemodynamically stable throughout his various previous ED visits and inpatient admission. Furthermore, laboratory investigations including complete blood count, electrolytes, renal, and hepatic parameters, were unremarkable. Cardiac work‐up, including cardiac markers, that is, cardiac Troponins, transthoracic echocardiography, 14 days holter monitoring were insignificant for myocardial damage. However, the only abnormality was his EKGs showed a coved pattern in V1‐V2 where an ascending and quick slope was present with a high take‐off ≥ 2 mm followed by concave down‐sloping ST, consistent with Brugada pattern type I (Figure 3).

## 4 Conclusion and Results (Outcome and Follow‐Up)

Cardiology team was consulted and an initial impression of a drug‐induced type I Brugada pattern was suspected based on his medication history and unremarkable laboratory and imaging studies. Since drug‐induced BrS is considered an intermediate‐risk factor for subsequent arrhythmic events and SCD in this population, he underwent a nuclear myocardial perfusion scan stress test. The test stopped due to dyspnea and fatigue. However, it did not demonstrate an arrhythmogenic pattern or ST segment changes. A transthoracic echocardiogram for evaluation of any structural heart disease showed an average left ventricle wall thickness, adequate left ventricular wall motion, and a 60% ejection fraction. He was referred for electrophysiological studies, and based on his telemetry monitoring, stress EKG and daily EKG findings of abnormal nonspecific ST segment changes, he was kept on aspirin 81 mg daily and atorvastatin 40 mg daily. He was seen by the neurologist and was started on topiramate for his migraine headaches. He was advised to follow general measures and avoid triggers like excessive caffeine, illicit drugs like cocaine, alcohol, energy drinks, lack of sleep, stressors or anxiety, and discontinuation of sumatriptan and amitriptyline. We provided him a list of medications to avoid triggering for BrS through a website link that is BrugadaDrugs.org. Upon post‐discharge follow‐up for 3–6 months, repeat EKGs did not reveal any Brugada‐like pattern and he also felt better symptomatically.

## 5 Discussion

BrS can occur secondary to genetic factors, electrolyte imbalances, drugs, fever, alcohol, medications, and other components yet to be discovered [1, 2]. It has an overall low prevalence of around 5 in 10,000 people worldwide, with a preponderance to men [2]. It can trigger life‐threatening dysrhythmias, including ventricular tachyarrhythmias, leading to SCD in patients. Literature illustrates the correlation with variants in almost 19 genes affecting the K^+^, Ca^2+^, and Na^+^ channels, specifically loss‐of‐function mutations of the SCN5A (sodium voltage‐gated channel alpha subunit 5) gene, which encodes the cardiac Na^+^ channel, leading to decreased myocardial sodium inflow currents, lowering the action potentials, changes in the action potential in the epicardium, and in the M cells, causing ST segment elevation and T‐wave inversion on EKG [3].

Patients can present with a vast symptomatology, including syncope, agonal breathing, palpitations, and dyspnea, which prompts further investigations [3]. Three EkG patterns can be observed in patients with BrS: Type 1 pattern, where a coved‐shaped ST‐segment elevation (STE) ≥ 2 mm and negative T‐wave occur in the right precordial leads; Type 2 pattern, with a saddleback morphology and STE ≥ 1 mm in precordial leads; and Type 3, STE < 1 mm with either a coved or saddleback morphology [4].

There are predictive scales to help with the diagnosis as part of patient evaluation. The Shanghai BrS score, where patients receive points for EKG findings, clinical findings, family history, and genetic results, provides a tool to differentiate between nondiagnostic, possible, probable, or definite BrS [5].

A thorough medical history is essential to identify precipitating factors, including medications, illicit drugs, excessive caffeine, alcohol, etc. Drugs known to induce BrS include those that mainly affect Na^+^ channel blockage, including antiarrhythmic drugs such as Pilsicainide, ajmaline, flecainide, calcium channel blockers (verapamil), beta‐blockers (isoproterenol) and psychotropic drugs, especially amitriptyline, which could also be a potential trigger in our patient, along with sumatriptan, selective serotonin reuptake inhibitors, specifically paroxetine, fluvoxamine, and lithium, which are scarce [6, 7]. There are some reports of BrS associated with supratherapeutic phenytoin levels and antihistamines (described in cases of diphenhydramine overdose) [8]. Sudden cardiac arrest has been reported in 7 cases with Propofol, which is a commonly used anesthetic in clinical settings [9].

Triptans such as Sumatriptan bind mainly to 5‐HT1B and 5‐HT1D receptors within cerebral blood vessels, leading to vasoconstriction in addition to inhibition of the release of neurogenic inflammatory mediators such as calcitonin gene‐related peptide [10]. Cases of sumatriptan leading to arrhythmias have been reported previously, including cases of polymorphic ventricular tachycardia, VF, and atrial fibrillation [11]. However, only a few cases of sumatriptan unmasking Type I Brugada pattern have been reported [12]. *Carbonara* et al. studied sumatriptan effects at a cellular level and its role in voltage‐gated sodium channel blockage on peripheral neurons as part of analgesia management. They demonstrated that sumatriptan can block sodium channels only at very high, supratherapeutic concentrations. Therefore, implying that the sumatriptan actions are not limited to the selective blockage of HT receptors, Instead they can potentially indirectly affect other receptors that have yet to be discovered [13].

For triptans, the cutoff overuse is ≥ 10 days/month for at least 3 months [14]; therefore, we can assume, in our patient, that medication overuse was taking place, which might have led to side effects, including BrS, and later it was triggered by amitriptyline. Medication reconciliation appears as a way of mitigating such problems since it can minimize adverse drug events. If a drug‐induced BrS is suspected, discontinuation of the causative agent is the first treatment approach in most cases, and ICD implantation in a specific eligible number of patients.

## 6 Conclusion/Aim

BrS results from a complex interaction between genetics and external factors, including medications. MOH is a global concern. In patients prescribed triptans or other arrhythmogenic drugs for migraine headaches abortive therapy, screening for cardiovascular risk factors, medication reconciliation, and patient education regarding side effects are highly encouraged to reduce the risk of potentially fatal cardiac arrhythmias.

## Author Contributions

**Phool Iqbal:** conceptualization, supervision, validation, visualization, writing – review and editing. **Saba Nabavi Monfared:** visualization, writing – review and editing. **Daniel Hernan Sacoto:** conceptualization, writing – original draft. **Mustafa Bilal Ozbay:** writing – review and editing. **Wael Abdelmottaleb:** writing – review and editing. **Valentina Turbay Caballero:** writing – review and editing. **Muhammad Khalid Tahir:** visualization, writing – review and editing. **Savi Mushiyev:** supervision, visualization, writing – review and editing.

## Ethics Statement

The patients have consented to publish this case. The study is conducted ethically in accordance with the World Medical Association Declaration of Helsinki.

## Consent

The patient/family has consented to publish this case, and written/verbal informed consent has been taken from the patient/family. The study is conducted ethically in accordance with the World Medical Association Declaration of Helsinki.

## Conflicts of Interest

The authors declare no conflicts of interest.

## Data Availability

Authors confirm that all relevant data or information are included in the article and is available via the open access platform of this journal.
